# Effects of Ambient Temperature on the Performance and Thermoregulatory Responses of Commercial and Crossbred (Brazilian Piau Purebred Sires × Commercial Dams) Growing-Finishing Pigs

**DOI:** 10.3390/ani11113303

**Published:** 2021-11-19

**Authors:** Vinícius Eduardo Moreira, Renata Veroneze, Alípio dos Reis Teixeira, Lorena Duarte Campos, Lais Fernanda Lopes Lino, Gabryele Almeida Santos, Bruno Alexander Nunes Silva, Paulo Henrique Reis Furtado Campos

**Affiliations:** 1 Animal Science Postgraduate Program, Department of Animal Science, Universidade Federal dos Vales do Jequitinhonha e Mucuri, Diamantina 39100-000, MG, Brazil; vinicius.e.moreira@ufv.br (V.E.M.); alipiodosreisteixeira@outlook.com (A.d.R.T.); 2 Department of Animal Science, Universidade Federal de Viçosa, Viçosa 36570-900, MG, Brazil; renata.veroneze@ufv.br (R.V.); lorena.duarte@ufv.br (L.D.C.); lais.lino@ufv.br (L.F.L.L.); gabryele.santos@ufv.br (G.A.S.); 3 Institute of Agricultural Sciences, Department of Animal Science, Universidade Federal de Minas Gerais, Montes Claros 39404-547, MG, Brazil; brunosilva@ufmg.br

**Keywords:** adaptation, genotype, heat stress, native breeds, thermotolerance

## Abstract

**Simple Summary:**

Physiological responses to heat stress are affected by breed. Therefore, crossbreeding genetically improved lines with tropically adapted breeds of pigs may be a strategy to attenuate the impact of high ambient temperatures on pig production. Although some studies have evaluated thermotolerance in tropically adapted breeds, it is not yet clear to which extent improved tolerance to heat stress is a consequence of a greater ability to equilibrate thermogenesis and thermolysis, or if it is a consequence of decreased growth performance. Although there was no interaction for performance, thermoregulatory responses, and blood parameters, our results evidenced that ambient temperature effects on carcass parameters were modulated by the pigs’ genotype. Because protein deposition significantly decreased in response to high ambient temperature in commercial pigs, and was not affected by ambient temperature in Piau crossbred pigs, our study suggests increased thermotolerance of Piau crossbred pigs.

**Abstract:**

The study aimed at evaluating the effects of high ambient temperature (HT: 30 °C) on the thermoregulatory responses and performance of commercial and Piau crossbred (Brazilian Piau breed sires × commercial genotype dams) growing pigs. Commercial and Piau crossbred pigs were reared under thermoneutral (TN: 22 °C) or HT conditions during a 14-day experimental period. Feeding (daily) and animals (beginning and end) were weighted to obtain performance parameters. Skin and rectal temperatures, respiratory rate, and blood parameters were also measured. At the end of the trial (day 15), the animal’s backfat thickness (BF) and loin eye area (LEA) were measured. No interaction (*p* > 0.05) between the genetic group and ambient temperature was observed for any performance trait. Irrespective of ambient temperature, Piau crossbred pigs had a similar feed intake (ADFI, 2615 g/day, on average; *p* > 0.05), lower daily weight gain (ADG, −234 g/day; *p* < 0.01), and a higher feed conversion ratio (FCR, +0.675 g/g; *p* < 0.01). There was interaction (*p* = 0.01) between genotype and ambient temperature for the LEA that decreased significantly in response to HT in commercial pigs (−6.88 cm^2^) and did not differ in response to ambient temperature in Piau crossbred pigs (29.14 cm^2^, on average; *p* > 0.05). Piau crossbred pigs had greater BF (+7.2 mm; *p* < 0.01) than commercial pigs. Regardless of the genetic group, exposure of pigs to HT resulted in decreased ADFI (−372 g/day; *p* < 0.01), ADG (−185 g/day; *p* < 0.01), and a higher FCR (+0.48 g/g; *p* = 0.01). Ambient temperature did not affect lipid deposition. Pigs at HT had an increased respiratory rate (+38 bpm; *p* < 0.01) and a long-lasting increase in skin and rectal temperatures compared to TN pigs. Total concentrations of triiodothyronine (T_3_) and thyroxine (T_4_) were not affected by ambient temperature in commercial pigs, whereas Piau crossbred pigs kept at 30 °C had a transient decrease in both hormones at day 2 (*p* < 0.01). Serum cortisol concentrations were not affected (*p* > 0.05) by genotype nor ambient temperature. In summary, Piau crossbred pigs had lower efficiency using nutrients for growth in association with increased lipid deposition when compared to commercial pigs. In response to HT, commercial pigs had a decreased LEA, whereas no effect was observed for Piau crossbred pigs. Apart from that, commercial and Piau crossbred pigs had a similar magnitude of thermoregulatory responses activation in response to HT, evidencing their innate survival-oriented function.

## 1. Introduction

Ambient temperatures above the thermoneutral zone for growing pigs (15 to 25 °C) [1] can be a source of extensive financial loss in production systems by affecting the performance and welfare of pigs [2,3,4]. Moreover, Renaudeau et al. [5] reported that physiological responses to heat stress are affected by the breed of pig. Thus, selecting thermotolerant animals might be a more consistent production strategy since it produces a permanent and intrinsic animal adaptation with no transient extra costs [6].

According to Carabaño et al. [7], heat-tolerant animals are those that, under high ambient temperature, maintain homeothermy by balancing heat production and dissipation without productive and reproductive losses. In accordance, Teixeira et al. [8] suggested in a previous study a greater heat-tolerance in Piau pigs than commercial pigs, due to the decreased magnitude of feed intake impairment and preserved feed conversion rate of the native Brazilian breed at high ambient temperatures. In addition, Rosé et al. [9] and Gourdine et al. [3] showed genetic variation in the thermotolerance of pigs, suggesting an improvement in this trait by genetic selection.

The Brazilian native purebred Piau pig is a lard-type breed characterized by adaptability to adverse environmental conditions and resistance to diseases [10,11]. However, besides a lower productive performance and muscularity, the Piau pig has more subcutaneous and intramuscular fat compared to current commercial lines [12,13]. This study was performed under the hypothesis that the crossbreeding of non-selected local pig breeds with commercial genotypes would improve the thermotolerance of progeny pigs. Thus, the study aimed at evaluating the effects of ambient temperature on the performance and thermoregulatory responses of commercial and crossbred (Brazilian Piau purebred sires × commercial dams) growing-finishing pigs.

## 2. Materials and Methods

All animal procedures followed the Brazilian Legislation on Animal Experimentation and Welfare. The Animal Care and Use Committee of the Universidade Federal de Viçosa (CEUAP), MG, Brazil, approved the experimental protocols (protocol code 27/2018).

### 2.1. Experimental Design and Pig Management

The research was carried out at the Pig Breeding Research Unit of the Universidade Federal de Viçosa and included thirty-five barrows. The research trial was conducted in a completely random design under 2 × 2 factorial schemes consisting of two ambient temperature conditions, thermoneutral (22 °C, TN) and high (30 °C, HT) ambient temperatures, and two genetic groups, commercial (commercial genotype sires × commercial genotype dams) and Piau crossbred (Brazilian Piau purebred sires × commercial genotype dams) pigs. The trial was conducted using nine and ten Piau crossbred barrows at TN and HT conditions, respectively, and eight commercial barrows in each ambient condition. Animals were evaluated at a similar initial body weight (BW) and age that consisted of 71.2 ± 1.94 kg BW and 108 ± 7 days old for commercial genotype pigs, and 68.9 ± 1.93 kg BW and 112 ± 4 days old for Piau crossbred pigs. Piau purebred sires used for mating originated and belonged to the Piau Breed Genetic Conservation Program, conducted since 1998 at the Universidade Federal de Viçosa.

Pigs were individually housed according to their allocation (TN or HT) in climatic-controlled rooms in suspended metal pens (0.80 × 1.60 m) with slatted floors, equipped with nipple drinkers and semi-automatic feeders. In each climatic-controlled room, automated climate systems composed of electric heaters, infrared lamps, and a chiller cooling system maintained temperature control. The room’s temperature was monitored by a data logger (Klimalogg Pro, TFA Dosmann, Werthein, Germany). Relative humidity was not controlled in the study.

The experimental period lasted 21 days, which consisted of a 7-day adaptation period (from days −7 to −1), wherein pigs were kept at 24 °C as a precaution to avoid any overreaction and/or severe discomfort in response to ambient temperature transition from the adaptation to experimental phase, and a subsequent 14-day experimental period (from days 1 to 14), wherein pigs were kept at 22 °C (TN) or 30 °C (HT). Feed and water were provided *ad libitum* throughout the experiment, including during the adaptation and experimental periods. Irrespective of genotype, pigs were fed the same corn and soybean meal-based diet formulated to meet or exceed the nutritional requirements of commercial genotype barrows with medium performance reared under thermoneutral conditions, according to Rostagno et al. [14]. The diet provided 3280 kcal of ME/kg and 150 g of CP/kg.

### 2.2. Measurements and Sampling Procedures

Provided feed and leftovers were recorded daily to obtain the average daily feed intake (ADFI; g/day). In addition, to measure the average daily gain (ADG; g/day) and the feed conversion rate (FCR; g/g), pigs were weighed without fasting in the morning at days 1 and 15. Skin and rectal temperature (RT) and respiratory rate (RR) were measured at days −4, −2, 1, 2, 3, 5, 7, 9, 11 and 13 at 3:00 PM. Measurements performed during the adaptation periods aimed to avoid any influence of the procedures in the observations.

**Figure 1:**
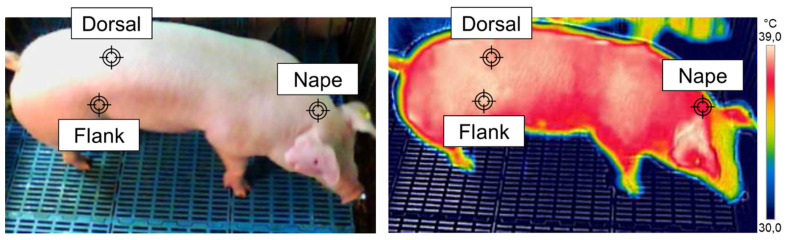
Graphical representation of nape, dorsal and flank skin temperatures measured through thermographic analysis in pigs reared under thermoneutral and high ambient temperatures.

Respiratory rate was visually determined by counting flank movements over 15 s, then the values were calculated for one minute, as previously described [15,16,17]. Nape, dorsal and flank skin temperature measurements were performed according to Teixeira et al. [8]. Briefly, images were taken using an infrared camera (C2 FLIR; FLIR Vision Systems, AB, Sweden; 0.95 emissivity and ± 2 °C of accuracy) at the level of the backline, 1.0 m away from the animal body, allowing pigs to be fully framed in the image (Figure 1). The obtained images were processed using FLIR tools software (Thermo Cam Research Pro 2.7; FLIR Vision Systems, AB, Linköping, Sweden). Finally, RT was measured using a clinical digital thermometer (Multilaser Hc070, São Paulo, Brazil) with an accuracy of ±0.1 °C.

At days 2, 9 and 15, between 7:00 and 8:00 AM, blood samples of all animals were collected via the orbital sinus bleeding technique, and serum was separated from the coagulated blood by centrifugation at 3000 rpm at 4 °C (refrigerated centrifuge) for 10 min. Serum samples were analyzed for total circulating triiodothyronine (T_3_), thyroxine (T_4_), and cortisol by the direct chemiluminescence method (Atellica IM Analyzer).

At the end of the experimental period (day 15), all pigs were slaughtered. Pre-harvest handling and slaughtering were carried out according to animal welfare regulations. After skinning and evisceration, the right halves of the carcasses were stored in a cold room at 4 °C for 24 h. After this period, a transversal cut in the right-half carcass at the 10th rib region was performed to assess backfat thickness (BF) and the loin eye area (LEA), according to the method described in our previous study [8]. Briefly, BF was measured using a digital caliper (Mitutoyo Corp, Kanogawa, Japan), and the LEA was measured by tracing the *longissimus dorsi* muscle outline onto transparent paper, then measuring the area through ImageJ software (version 1.51, National Institutes of Health, Bethesda, MD, USA).

### 2.3. Data Statistical Analyses

Performance and carcass parameters were analyzed using the PROC GLM model of SAS [18], considering the fixed effects of the genetic group, ambient temperature, and their interaction. The following statistical model was used:

Y_ijk_ = μ + X + G_i_ + Ta_j_ + (G × Ta)_ij_ + ε_ijk_,

where Y_ijk_ is the observation; μ is the general constant; X is the covariate (initial body weight); G_i_ is the effect of genetic group (commercial or Piau crossbred); Ta_j_ is the fixed effect of ambient temperature (22 or 30 °C); (G × Ta)_ij_ is the interaction between genetic group and ambient temperature; and ε_ijk_ is the random error. The experimental unit was represented by the individual pig.

Thermoregulatory responses and blood parameters data were analyzed using the PROC MIXED model of SAS [18], considering the fixed effects of genetic group, ambient temperature, day of measurement, and their interactions. The following statistical model was used:

Y_ijkl_ = μ + G_i_ + D_j_ + Ta_k_ + (G × D × Ta)_ijk_ + (G × D)_ij_ + (G × Ta)_ik_ + (D × Ta)_jk_ + ε_ijkl_,

where Y_ijk_ is the observation; μ is the general constant; G_i_ is the fixed effect of genetic group (commercial or Piau crossbred); D_j_ is the fixed effect of measurement day; Ta_k_ is the fixed effect of ambient temperature (22 or 30 °C); (G × D × Ta)_ijk_ is the interaction between genetic group, day of measurement, and ambient temperature; (G × D)_ij_ is the interaction between genetic group and day of measurement; (G × Ta)_ik_ is the interaction between genetic group and ambient temperature; (D × Ta)_jk_ is the interaction between day of measurement and ambient temperature; and ε_ijk_ is the random error. The repeated measurement option was used with a compound symmetry covariance structure to account for animal effect over experimental days. Adjusted means were compared by a Tukey test, and the effects were considered significant if *p*-value < 0.05.

## 3. Results

Because of health problems and excessive feed spillage, data from five animals subjected to high ambient temperature (two commercial and three crossbred pigs) were not considered in the analysis. During the experimental period, the ambient temperature in thermoneutral and high ambient temperature climate-controlled rooms averaged 22.6 ± 1.8 °C and 29.4 ± 1.8 °C, respectively. These ambient temperature values were in accordance with the objectives of the experiment.

### 3.1. Growth Performance and Carcass Parameters

Piau crossbred pigs had lower initial body weight (BW) than commercial pigs at the beginning of the trial (−2.15 kg; *p* < 0.01). Therefore, initial BW was considered as a covariate for all performance and carcass variables. Table 1 shows the effects of ambient temperature on the performance and carcass parameters of commercial and Piau crossbred pigs. No interaction (*p* > 0.05) between genetic group and ambient temperature was observed for any performance trait. Irrespective of ambient temperature, Piau crossbred pigs had similar ADFI (2615 g/day, on average; *p* = 0.82), lower ADG (−234 g/day; *p* < 0.01), and a higher FCR (+0.675 g/g; *p* < 0.01) than commercial pigs. Regarding genetic group, both genotypes exposed to HT exhibited lower ADFI (−372 g/day; *p* < 0.01) and ADG (−185 g/day; *p* < 0.01), and a higher FCR (+0.48 g/g; *p* = 0.01) than pigs in TN. No interaction (*p* > 0.05) between genotype and ambient temperature was found for backfat thickness. However, the interaction was significant (*p* = 0.01) for the LEA, which decreased significantly in response to HT in commercial pigs (−6.88 cm^2^; *p* < 0.05) and did not differ in response to ambient temperature in Piau crossbred pigs (29.14 cm^2^, on average; *p* > 0.05). Regardless of ambient temperature, Piau crossbred pigs had greater backfat thickness (+7.2 mm; *p* *<* 0.01) and lower loin eye area (−6.82 cm^2^; *p* < 0.01) than commercial pigs. Ambient temperature did not affect lipid deposition.

### 3.2. Thermoregulatory Responses and Blood Parameters

**Figure 2:**
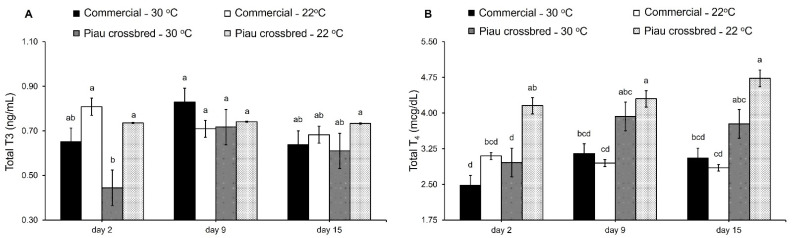
Serum triiodothyronine (**A**) and thyroxine (**B**) concentrations of commercial and Piau crossbred (Brazilian Piau purebred sires × commercial dams) pigs as a function of ambient temperature. Each vertical bar is the standard error of the mean. Within each graphic, least square means with different letters differ (*p* < 0.05).

Ambient temperature effects on the thermoregulatory responses of commercial and Piau crossbred pigs are shown in Table 2. Regardless of ambient temperature, commercial pigs had greater dorsal (+0.4 °C; *p* < 0.01) and flank (+0.4 °C; *p* < 0.01) temperatures, whereas nape (36.2 °C, on average; *p* > 0.05) and rectal temperatures (39.5 °C, on average; *p* > 0.05) did not differ. The exposure of pigs to high ambient temperature resulted in increased nape (+1.6 °C; *p* < 0.01), dorsal (+2.0 °C; *p* < 0.01), flank (+1.7 °C; *p* < 0.01), and rectal temperatures (+0.4 °C; *p* < 0.01), as well as an increased respiratory rate (+38 bpm; *p* < 0.01), compared to thermoneutral conditions. Interaction between genotype, ambient temperature, and day was observed for circulating levels of total T_3_ and T_4_ (Figure 2). Even with the increase in ambient temperature, T_3_ and T_4_ serum concentrations remained relatively steady in commercial pigs, whereas Piau crossbred pigs kept at HT had lower T_3_ and T_4_ concentrations at day 2 (*p* < 0.01) than Piau crossbred pigs at TN. Neither genotype, ambient temperature, nor their interaction affected (*p* > 0.05) serum cortisol concentrations.

## 4. Discussion

Among strategies to attenuate the negative effects of high ambient temperatures in pig production, the crossbreeding of genetically improved lines with tropically adapted breeds of pigs has been suggested as a strategy to select thermotolerant genotypes [19]. Piau crossbred pigs had lower growth performance (body weight gain and final body weight) than commercial pigs. Part of these results may be explained by the higher fat deposition and lower muscularity (LEA) of Piau than commercial pigs. In agreement, Veloso et al. [20] reported the decreased performance of Piau crossbred pigs compared with Duroc and Piétrain crossbred pigs. Thus, our findings are in accordance with the previously stated decreased productive performance and high subcutaneous fat of Piau crossbred pigs [12]. These results were expected since Brazilian native purebred Piau pigs are recognized for their high-fat deposition [13] and lower growth performance [21]. Teixeira et al. [8] have reported a reduction of more than 300 g/day in weight gain, an increase of more than 1.5 g/g in feed conversion, and a two-fold higher backfat thickness of Piau purebred pigs compared to commercial genotypes.

When the ambient temperature exceeds the upper critical limit (25 °C), sensible heat exchanges lose effectiveness, giving way to latent heat loss such as respiratory evaporation. This thermoregulatory response is dependent on ambient vapor pressure and can increase energy and nutrient demand, resulting in negative effects on growth performance [22,23]. Concomitantly, pigs reduce feed intake at high ambient temperatures as an effective mechanism to reduce metabolic heat production associated with the thermic effect of feeding [4,5,22]. Therefore, fewer nutrients would be available for growth, impairing the overall production performance, which justifies the decreased daily weight gain (21%) in heat-stressed pigs. Likewise, Santos et al. [17] and Teixeira et al. [8] also verified a reduced feed intake and weight gain in pigs raised under hot conditions, even in different genetic groups (genotype commercial and Piau purebred pigs, respectively), corroborating our findings. Furthermore, no matter the genotype, high temperature resulted in a worse feed efficiency, suggesting that the decreased growth performance at high temperatures reported in our study was not exclusively associated with a reduction in feed intake, but was probably due to the decreased capacity of the animals to use or allocate nutrients for growth.

As reported in other studies, the increased energy requirement for maintenance due to panting [22] and increased chemical reaction rates [24] occurs in pigs exposed to high ambient temperatures.

According to our results, ambient temperature effects on carcass parameters were modulated by the pigs’ genotype. The loin eye area was significantly decreased in response to high ambient temperature in commercial pigs, whereas it was not affected by ambient temperature in Piau crossbred pigs. Because protein deposition is directly associated with metabolic heat production [25,26], its reduction at high ambient temperatures may be interpreted as a strategy of commercial pigs to attenuate negative effects of heat stress. Because this response was not observed in Piau crossbred pigs, increased thermotolerance of this genotype could be suggested. The fact that lipid deposition was not affected by ambient temperature could be associated with the increased energetic cost of protein compared to adipose tissue deposition. For instance, although 2 ATP are necessary to deposit lipid, a peptide bond synthesis from amino acids needs at least 5 ATP [26,27]. This suggested that commercial pigs prioritized inhibiting proteogenesis over lipogenesis at high ambient temperatures to thermoregulate. Similarly, Fraga et al. [26] reported that pigs with a greater proportion of Piétrain genes had an increased susceptibility to heat stress due to their presumed increased metabolic heat production associated with a greater growth rate and lean tissue deposition.

As both genetic groups had higher skin temperatures at 30 °C than at 22 °C, it is assumed that increased peripheral circulation might be associated with cutaneous vasodilation to aid heat loss to the environment by the regular pathways, mainly conduction and convection [4,19]. Interestingly, irrespective of ambient temperature crossbred Piau pigs had lower dorsal and flank skin temperatures than commercial pigs, which probably results from the greater insulation effect of the backfat. As the ambient temperature rises above the thermoneutral zone, animals must rely on evaporative heat losses to dissipate heat [15]. Because pigs have a limited number of functional sweat glands [28], their main evaporative pathway is through the increase in respiratory evaporation [16,29], which can explain the 53% increase in respiratory rate of pigs at high ambient temperature conditions.

Rectal temperature is not a heat stress response but rather a physiological indicator of core temperature [8]. Because of the complexity and fine control of this physiological condition, even a minimal variation demands caution and suggests a physiological disturbance. Despite the activation of thermoregulatory responses (behavioral, physiological, and metabolic adjustments), increased rectal temperature in commercial and Piau crossbred pigs (+0.4 °C) suggests an incapacity of pigs to avoid hyperthermia in the experimental conditions. Increased rectal temperature in pigs exposed to high ambient temperature has been consistently described in previous studies [5,8,16], evidencing the susceptibility of pigs to such conditions.

In terms of blood parameters, heat acclimation involves complex interactions between the thermoregulatory and endocrine systems [30]. The thyroid gland plays a crucial role in heat acclimation since it releases T_3_ and T_4_ hormones, which stimulate the metabolic rate and heat production by activating decoupling oxidative phosphorylation in mitochondria [16,31]. Therefore, heat acclimation comprises decreased thyroid gland activity. Accordingly, Kouba et al. [32] reported a decrease in thyroid gland weight by 23% at 31 °C compared to pair-fed pigs reared at 20 °C. In addition, Zhang et al. [33] reported lower T_3_ and T_4_ levels in 25 kg BW pigs when ambient temperature increased from 18 °C to 32 °C. The current study shows that high ambient temperature results in an overall decrease in thyroid hormone concentrations. However, only Piau crossbred pigs were affected by the ambient temperature on day 2, which suggests that the thermoregulatory mechanism modifies according to the genetics of the pig. The reduction in serum concentrations of thyroid hormones is an acclimation mechanism of pigs to avoid extra heat load, decreasing basal metabolic rate and thus heat production [34].

Cortisol is a glucocorticoid of the hypothalamic–pituitary–adrenal axis extensively used as an indicator of non-specific stress responses [34]. According to Campos et al. [34], cortisol responses may vary according to the magnitude and duration of heat stress extent (acute or chronic). In the current study, although not significant, serum cortisol concentrations were numerically lower at 30 °C than at 22 °C irrespective of the pigs’ genotype. Accordingly, in moderate chronic heat stress conditions, cortisol levels tend to be reduced to avoid extra heat production related to catabolic processes, as previously described by Campos et al. [16], Heo et al. [35], and Kim et al. [36].

## 5. Conclusions

In summary, Piau crossbred pigs had lower efficiency using nutrients for growth in association with increased lipid deposition when compared to commercial pigs. In response to high ambient temperature, commercial pigs had decreased lean tissue deposition, whereas no effect of ambient temperature was observed for Piau crossbred pigs, suggesting increased thermotolerance. Apart from that, commercial and Piau crossbred pigs had a similar magnitude of thermoregulatory responses activation in response to heat stress, evidencing its innate survival-oriented function.

## Figures and Tables

**Table 1:** Ambient temperature effects on performance and carcass parameters of commercial and crossbred (Brazilian Piau purebred sires × commercial dams) pigs.

	Commercial		Piau Crossbred		RMSE ^1^	Statistical Analysis		
	22 °C	30 °C	22 °C	30 °C		Gen	Temp	Gen × Temp
Number of pigs	8	6	9	7				
Performance parameters								
Initial body weight (kg)	71.8	71.1	70.3	68.3	2.1	<0.01	<0.01	0.62
Average daily feed intake (g/day)	2933	2512	2669	2346	287	0.82	<0.01	0.35
Average daily gain (g/day)	1015	773	724	596	193	<0.01	<0.01	0.39
Feed conversion rate (g/g)	2.96	3.55	3.75	4.11	0.87	<0.01	0.01	0.86
Final body weight (kg)	78.9	76.5	75.4	72.4	1.4	<0.01	<0.01	0.39
Carcass parameters								
Backfat thickness (mm)	17.3	16.6	24.5	23.8	4.4	<0.01	0.67	0.99
Loin eye area (cm^2^)	39.40 ^a^	32.52 ^b^	29.08 ^b^	29.20 ^b^	3.16	<0.01	0.01	0.01

^1^ Root-Mean-Square Error. ^a,b^ within a row, least-square means with different superscript are statistically different (*p* < 0.05).

**Table 2:** Ambient temperature effects on thermoregulatory responses and blood parameters of commercial and crossbred (Brazilian Piau purebred sires × commercial dams) pigs.

	Commercial		Piau Crossbred		RSD ^1^	Statistical Analysis ^2^			
	22 °C	30 °C	22 °C	30 °C		Gen	Temp	Gen × Temp	Gen × Temp × Day
Number of pigs	8	6	9	7					
Skin surface temperature (°C)									
Nape	35.6	36.9	35.3	37.1	0.8	0.49	<0.01	0.08	0.18
Dorsal	35.8	37.7	35.3	37.4	0.6	<0.01	<0.01	0.31	0.07
Flank	36.3	37.9	35.8	37.6	0.7	<0.01	<0.01	0.27	0.16
Rectal temperature (°C)	39.4	39.8	39.2	39.6	0.2	0.06	<0.01	0.89	0.08
Respiratory rate (bpm ^3^)	71	105	69	110	19	0.83	<0.01	0.48	0.12
Blood parameters									
T_3_ (ng/mL)	0.73	0.71	0.74	0.59	0.13	0.07	<0.01	0.05	0.01
T_4_ (mcg/dL)	2.97	2.89	4.40	3.55	0.37	<0.01	0.03	0.07	<0.01
Cortisol (mcg/dL)	3.78	3.08	3.52	3.11	1.40	0.84	0.34	0.80	0.72

^1^ Residual Standard Deviation. ^2^ The observed interaction (*p* < 0.05) between Gen × Temp × Day for T_3_ and T_4_ circulating levels are presented in Figure 2. ^3^ Breaths per minute.

## Data Availability

The data presented in this study are available on request from the corresponding author.

## References

[1] Federation of Animal Sciences Societies (2010). Guide for the Care and Use of Agricultural Animals in Research and Teaching.

[2] Kim K.S., Seibert J.T., Edea Z., Graves K.L., Kim E.S., Keating A.F., Baumgard L.H., Ross J.W., Rothschild M.F. (2018). Characterization of the acute heat stress response in gilts: III. Genome-wide association studies of thermotolerance traits in pigs. J. Anim. Sci..

[3] Gourdine J.L., Riquet J., Rosé R., Poullet N., Giorgi M., Billon Y., Renaudeau D., Gilbert H. (2019). Genotype by environment interactions for performance and thermoregulation responses in growing pigs. J. Anim. Sci..

[4] Mayorga E.J., Renaudeau D., Ramirez B.C., Ross J.W., Baumgard L.H. (2019). Heat stress adaptations in pigs. Anim. Front..

[5] Renaudeau D., Huc E., Noblet J. (2007). Acclimation to high ambient temperature in Large White and Caribbean Creole growing pigs. J. Anim. Sci..

[6] Osei-Amponsah R., Chauhan S.S., Leury B.J., Cheng L., Cullen B., Clarke I.J., Dunshea F.R. (2019). Genetic selection for thermotolerance in ruminants. Animals.

[7] Carabaño M.J., Ramón M., Menéndez-Buxadera A., Molina A., Díaz C. (2019). Selecting for heat tolerance. Anim. Front..

[8] dos Reis Teixeira A., Veroneze R., Moreira V.E., Campos L.D., Raimundi S.C.J., Campos P.H.R.F. (2021). Effects of heat stress on performance and thermoregulatory responses of Piau purebred growing pigs. J. Therm. Biol..

[9] Rose R., Gilbert H., Loyau T., Giorgi M., Billon Y., Riquet J., Renaudeau D., Gourdine J.L. (2017). Interactions between sire family and production environment (temperate vs. tropical) on performance and thermoregulation responses in growing pigs. J. Anim. Sci..

[10] da Mariante S.A., Castro S.T.R., Albuquerque M.S.M., Paiva S.R., Germano J.L. (2003). Pig biodiversity in Brazil. Arch. Zootec..

[11] Sollero B.P., Paiva S.R., Faria D.A., Guimarães S.E.F., Castro S.T.R., Egito A.A., Albuquerque M.S.M., Piovezan U., Bertani G.R., Mariante A.D.S. (2009). Genetic diversity of Brazilian pig breeds evidenced by microsatellite markers. Livest. Sci..

[12] Serão N.V.L., Veroneze R., Ribeiro A.M.F., Verardo L.L., Braccini Neto J., Gasparino E., Campos C.F., Lopes P.S., Guimarães S.E.F. (2011). Candidate gene expression and intramuscular fat content in pigs. J. Anim. Breed. Gen..

[13] Veroneze R., Lopes P.S., Guimarães S.E.F., Guimarães J.D., Costa E.V., Faria V.R., Costa K.A. (2014). Using pedigree analysis to monitor the local Piau pig breed conservation program. Arch. Zootec..

[14] Rostagno H.S., Albino L.F.T., Hannas M.I., Donzele J.L., Sakomura N.K., Perazzo F.G., Saraiva A., de Abreu M.L.T., Rodrigues P.B., Oliveira R.D. (2017). Tabelas Brasileiras para aves e Suínos: Composição de Alimentos e Exigências Nutricionais.

[15] Renaudeau D., Kerdoncuff M., Anais C., Gourdine J.L. (2008). Effect of temperature level on thermal acclimation in Large White growing pigs. Animal.

[16] Campos P.H.R.F., Noblet J., Jaguelin-Peyraud Y., Gilbert H., Mormède P., de Oliveira Donzele R.F.M., Donzele J.L., Renaudeau D. (2014). Thermoregulatory responses during thermal acclimation in pigs divergently selected for residual feed intake. Int. J. Biometeorol..

[17] Santos L.S.D., Pomar C., Campos P.H.R.F., da Silva W.C., Gobi J.D.P., Veira A.M., Fraga A.Z., Hauschild L. (2018). Precision feeding strategy for growing pigs under heat stress conditions. J. Anim. Sci..

[18] Statistical Analysis Software (SAS) (2004). SAS/STAT 9.1 User’s Guide.

[19] Renaudeau D., Collin A., Yahav S., De Basilio V., Gourdine J.L., Collier R.J. (2012). Adaptation to hot climate and strategies to alleviate heat stress in livestock production. Animal.

[20] Veloso R.D.C., Duarte M.D.S., Saraiva A., Guimarães S.E.F., Chizzotti M.L., Camargo E.G., Lopes P.S. (2019). Effects of nutritional plans and genetic groups on performance, carcass and meat quality traits of finishing pigs. Food Sci. Technol..

[21] Silva H.T., Ferreira A.S., Veroneze R., Lopes P.S. (2019). Evaluation of Bayesian models for analysis of crude protein requirement for pigs of Brazilian Piau breed. Sci. Agric..

[22] Quiniou N., Dubois S., Noblet J. (2000). Voluntary feed intake and feeding behaviour of group-housed growing pigs are affected by ambient temperature and body weight. Livest. Prod. Sci..

[23] Renaudeau D., Gourdine J.L., St-Pierre N.R. (2011). A meta-analysis of the effects of high ambient temperature on growth performance of growing-finishing pigs. J. Anim. Sci..

[24] Baumgard L.H., Rhoads R.P. (2013). Effects of heat stress on postabsorptive metabolism and energetics. Annu. Rev. Anim. Biosci..

[25] Brown-Brandl T.M., Nienaber J.A., Xin H., Gates R.S. (2004). A literature review of swine heat production. Trans. ASAE.

[26] Fraga A.Z., Campos P.H.R.F., Da Silva W.C., Caetano R.P., Veira A.M., Dos Santos L.S., Hauschild L. (2019). Sequential feeding with high-fat/low-crude protein diets for two lines of growing-finishing pigs under daily cyclic high ambient temperature conditions. J. Anim. Sci..

[27] van Milgen J., Noblet J. (2003). Partitioning of energy intake to heat, protein, and fat in growing pigs. J. Anim. Sci..

[28] Renaudeau D., Leclercq-Smekens M., Herin M. (2006). Differences in skin characteristics in European (Large White) and Caribbean (Creole) growing pigs with reference to thermoregulation. Anim. Res..

[29] Huynh T.T.T., Aarnink A.J.A., Verstegen M.W.A., Gerrits W.J.J., Heetkamp M.J.W., Kemp B., Canh T.T. (2005). Effects of increasing temperatures on physiological changes in pigs at different relative humidities. J. Anim. Sci..

[30] Becker B.A., Klir J.J., Matteri R.L., Spiers D.E., Ellersiek M., Misfeldt M.L. (1997). Endocrine and thermoregulatory responses to acute thermal exposures in 6-month-old pigs reared in different neonatal environments. J. Therm. Biol..

[31] Silvestri E., Schiavo L., Lombardi A., Goglia F. (2005). Thyroid hormones as molecular determinants of thermogenesis. Acta Phys. Scand..

[32] Kouba M., Hermier D., Le Dividich J. (2001). Influence of a high ambient temperature on lipid metabolism in the growing pig. J. Anim. Sci..

[33] Zhang S., Gao H., Yuan X., Wang J., Zang J. (2020). Integrative Analysis of Energy Partition Patterns and Plasma Metabolomics Profiles of Modern Growing Pigs Raised at Different Ambient Temperatures. Animals.

[34] Campos P.H.R.F., Floc’h N.L., Noblet J., Renaudeau D. (2017). Physiological responses of growing pigs to high ambient temperature and/or inflammatory challenges. Rev. Bras. Zootec..

[35] Heo J., Kattesh H.G., Roberts M.P., Morrow J.L., Dailey J.W., Saxton A.M. (2005). Hepatic corticosteroid-binding globulin (CBG) messenger RNA expression and plasma CBG concentrations in young pigs in response to heat and social stress. J. Anim. Sci..

[36] Kim B.G., Lindemann M.D., Cromwell G.L. (2009). The effects of dietary chromium (III) picolinate on growth performance, blood measurements, and respiratory rate in pigs kept in high and low ambient temperature. J. Anim. Sci..

